# Pseudophakic hyperopia in nanophthalmic eyes managed by a posterior chamber implantable collamer lens

**DOI:** 10.4103/0301-4738.77014

**Published:** 2011

**Authors:** Kulin J Kothari, Prakash R Nayak, Bijal K Mehta

**Affiliations:** Department of Cataract & Refractive Surgery, Bombay City Eye Institute and Research Centre, 5 Babulnath Road, Mumbai - 400 007, Maharashtra, India

**Keywords:** Ametropia, implantable collamer lens, nanophthalmos

## Abstract

We report a case of a bilateral posterior chamber implantable collamer lens (ICL) implantation post-clear lens extraction, to reduce the residual hyperopia, in a patient with nanophthalmic eyes. A 30-year-old female patient, keen to reduce her dependency on glasses and contact lenses, came to our refractive surgery department. Her refractive error was +12.0 and +12.5 diopters in the right and left eye, respectively, with steep corneas on keratometry and a shallow anterior chamber depth. She underwent clear lens extraction with implantation of +35.0 D and +40.0 D IOL in the right eye and left eye, respectively. Her post-operative best-corrected visual acuity was 20/30 with +8.5 D in the right eye and +6 D in the left. She underwent bilateral ICL implantation. Postoperatively after 6 months, her unaided visual acuity was 20/30 in both eyes. In conclusion, ICL implantation can be considered to correct residual hypermetropic ametropia in pseudophakic eyes when other options have limitations.

Implantable collamer lenses (ICLs) (STAAR Surgicals, Monrovia, LA, USA) have been used the world over in the last few years, and have shown encouraging results for treatment of high myopia and hyperopia.[1–4] There are not many reports of ICLs used to correct pseudophakic ametropia.[5] We report a case of a bilateral ICL implantation post-clear lens extraction, to reduce the residual hyperopia, in both nanophthalmic eyes of one patient.

## Case Report

A 30-year-old female patient came to our refractive surgery department for spectacle/contact lens independence. Her best spectacle corrected visual acuity (BSCVA) was 20/30 with +12.0 diopters, and +12.5 diopters for the right and left eye, respectively. External examination and slit-lamp examination showed no obvious abnormality. Gonioscopy showed occludable angles. Dilated fundus was also within normal limits.

Her horizontal corneal diameters were 11.5 mm in both eyes. Keratometry (Bausch & Lomb, Rochester, NY, USA) values were 48.5 D at 150° and 49.0 D at 60° in the right eye and 48.75 at 180° and 47.75 at 90° in the left eye. Axial lengths measured by IOL Master (Carl Zeiss Meditec AG, Jena, Germany) were 16.99 mm and 17.17 mm in the right and left eye, respectively. The anterior chamber depth (ACD) by Orbscan (Bausch & Lomb) was 2.68 mm from endothelium in both eyes.

The IOL power calculation in the right eye with SRK-II was +35 D for postoperative residual emmetropia. We performed a clear lens extraction (uneventful clear corneal temporal phacoemulsification) with the implantation of a +35 D AcrySof Aspheric Natural IOL (Alcon Laboratories, Fort Worth, TX, USA) in the right eye. Her postoperative BSCVA was 20/30 with +8.5 D. For the left eye we used SRK-T, which gave an IOL power value of +43 D for the aimed postoperative emmetropia. Two weeks later, she subsequently underwent the same procedure in the left eye with the implantation of the highest available power +40 D AcrySof Aspheric single-piece IOL. Her postoperative BSCVA was 20/30 with +6.0 D.

Her postoperative keratometry values were 49.0 D at 137 and 48.1 D at 7° in both eyes. Her postoperative reading was 3.88 mm (ACD) as measured by Orbscan (Bausch & Lomb). After discussing the clinical situation with colleagues and proper counseling of the patient, we decided on ICL implantation.

After bilateral YAG iridotomies, she underwent bilateral ICL implantation (3 months after clear lens extraction) for the correction of her residual hyperopic errors (+16 D, ICH115V3 for the right eye and +11.5 D ICH110V3 for the left eye (STAAR Surgicals). The ICL powers were derived by the modified Holladay formula for phakic IOLs.[6] Postoperatively her unaided visual acuity was 20/30 in both eyes. She again followed up after a period of 6 months post-ICL implantation. Her unaided visual acuity remained 20/30 in both eyes. IOP was 12 mmHg in both eyes.

## Discussion

Nanophthalmos is an abnormal smallness in all dimensions of one or both eyes in the absence of other ocular defects.[7] Phacoemulsification in nanophthalmos has been associated with significant intraoperative risks like vitreous loss, aqueous misdirection, and uveal effusion, primarily due to an increased scleral thickness.[8] This can be circumvented by prophylactic sclerotomies. In experienced hands, however, it may also be quite uneventful and may not require prophylactic sclerotomies.[9]

IOL power calculation in hyperopic eyes is usually challenging. Since the ratio of the anterior to posterior segments changes with increasing hyperopia, SRK II formula becomes less reliable. Other formulae like SRK-T, Hoffer Q, and Holladay II offer more accuracy with shorter axial lengths.[10] In this particular case, the suggested power by SRK-T was about 43 D in both eyes, which would have probably left the patient with a residual refractive error of about +5 D at the spectacle plane. We observed that though SRK-T was more reliable than SRK-II, in this particular case, the overall line of management would have remained the same for the correction of residual hyperopia.

In this patient, a primary hyperopic ICL would have been technically challenging due to low ACD. We did not consider Lasik as her corneas were too steep and it was too high a refractive error to be totally corrected by Lasik.

ICLs can be used to correct ametropia after cataract surgery.[5] This is a useful and technically simpler option to reduce ametropia, considering possible risks associated with other options like piggyback IOLs.[11] IOL exchange in this case was not considered due to nonavailability of higher diopter IOL.

To conclude, ICL implantation can be considered to correct residual hypermetropic ametropia in psuedophakic eyes when other options have limitations.
